# Supplementary material for: HRS phosphorylation drives immunosuppressive exosome secretion and restricts CD8+ T-cell infiltration into tumors
Source: Nat Commun. 2022 Jul 14;13:4078. doi: 10.1038/s41467-022-31713-6 (PMC9283393; doi:10.1038/s41467-022-31713-6)
Supplement: Supplementary file 1 — Supplementary Information [file 41467_2022_31713_MOESM1_ESM.pdf]

Supplementary Information

HRS Phosphorylation Selectively Drives Immunosuppressive Exosome Secretion and Spatially Restricts CD8+ T cell Infiltration into Tumors

Lei Guan[1*], Bin Wu[1*], Ting Li[2*], Lynn A. Beer[3], Gaurav Sharma[4], Mingyue Li[2], Chin Nien Lee[2], Shujing Liu[2], Changsong Yang[1], Lili Huang[2], Dennie T. Frederick[5], Genevieve M. Boland[6], Guangcan Shao[7], Tatyana M. Svitkina[1], Kathy Q. Cai[8], Fangping Chen[9], Meng-Qiu Dong[7], Gordon B. Mills[10], Lynn M. Schuchter[4,11], Giorgos C. Karakousis[2], Tara C. Mitchell[4,11], Keith T. Flaherty[5], David W. Speicher[3], Youhai H. Chen[2], Meenhard Herlyn[3], Ravi K. Amaravadi[4], Xiaowei Xu[2], Wei Guo[1,✉]

[1]Department of Biology, School of Arts & Sciences, University of Pennsylvania, Philadelphia, PA19104, U.S.A.

[2]Department of Pathology and Laboratory Medicine, Perelman School of Medicine, University of Pennsylvania, Philadelphia, PA19104, U.S.A.

[3]Molecular & Cellular Oncogenesis Program, Wistar Institute, Philadelphia, PA19104, U.S.A.

[4]Abramson Cancer Center, Perelman School of Medicine, University of Pennsylvania, Philadelphia, PA19104, U.S.A.

[5]Division of Medical Oncology, Department of Medicine, Massachusetts General Hospital Cancer Center, Harvard Medical School, Boston, MA02114, U.S.A.

[6]Department of Surgical Oncology, Massachusetts General Hospital, Boston, Massachusetts, MA02114, U.S.A.

[7]National Institute of Biological Sciences, Beijing 102206, P. R. China.

[8]Histopathology Facility, Fox Chase Cancer Center, Philadelphia, PA19111, U.S.A.

[9]Histotechnology Facility, The Wistar Institute, Philadelphia, PA19104, U.S.A.

[10]Division of Oncological Science, School of Medicine and Knight Cancer Institute, Oregon Health & Science University, Portland, OR 97201, U.S.A

[11]Department of Medicine, Perelman School of Medicine, University of Pennsylvania, Philadelphia, PA 19104, U.S.A.

[*]Lei Guan, Bin Wu and Ting Li contributed equally to the work.

CORRESPONDENCE: Wei Guo, Department of Biology, University of Pennsylvania, Philadelphia, PA 19104. Email: guowei@sas.upenn.edu

Table of Contents

Supplementary Figure 1 3
Supplementary Figure 2 4
Supplementary Figure 3 5
Supplementary Figure 4 6
Supplementary Figure 5 7
Supplementary Figure 6 8
Supplementary Figure 7 9
Supplementary Figure 8 10
Supplementary Figure 9 11
Supplementary Figure 10 12
Supplementary Figure 11 13
Supplementary Figure 12 14
Supplementary Figure 13 15
Supplementary Figure 14 16
Supplementary Table 1 17

Supplementary Fig. 1

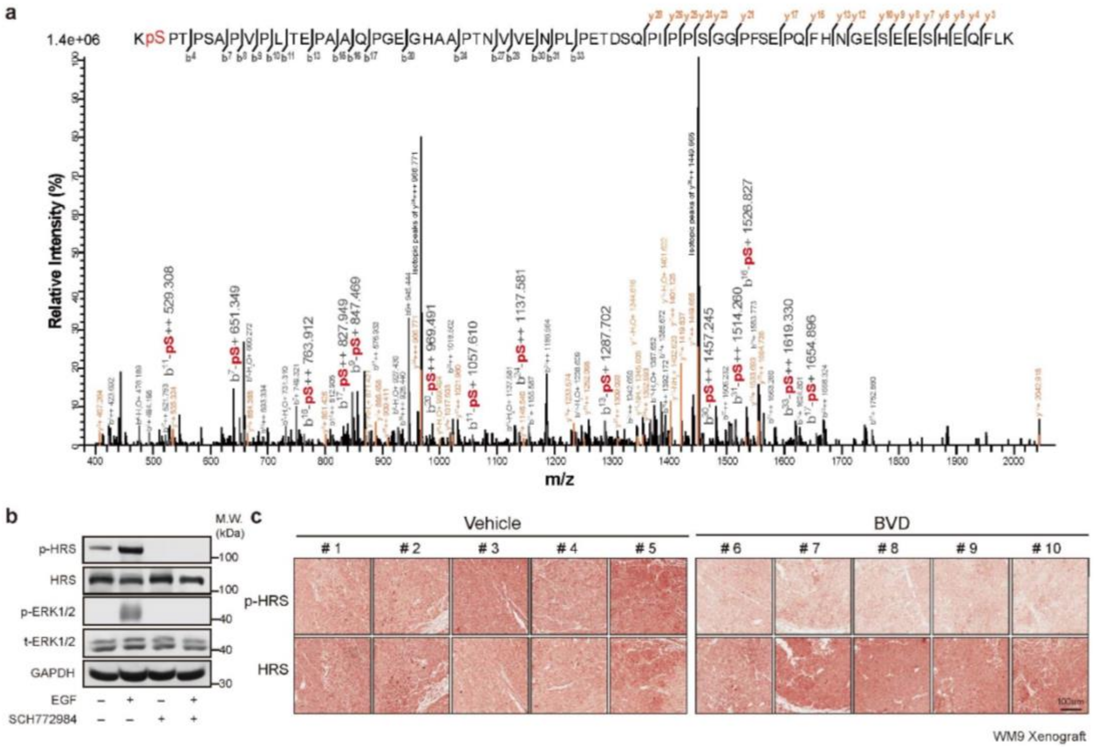

Supplementary Fig. 1. ERK1/2 phosphorylates HRS at Serine 345.

a, Annotated MS2 spectra identifying an HRS peptide phosphorylated at S345. Bold pS labels denote fragment ions containing a phosphoserine (pS) residue (red) (i.e.b4-b33). See *METHODS* for details. **b**, The HRS^{S345} phospho-peptide antibody recognized endogenous HRS in WM9 cells treated with EGF. HRS from cells treated with SCH772984 was not recognizable by this antibody. As a control, EGF treatment also activated ERK as shown by its phosphorylation (“pERK1/2”). Total ERK levels were also shown (“t-ERK”). GAPDH was used as a loading control. **c**, Representative IHC staining of p-HRS^{S345} and total HRS from WM9 xenografts established in *Rag2*$^{-/-}$ mice treated with vehicle control or BVD-523 (n=5). The upper and lower panels show the same areas of tumor tissues stained for total HRS and p-HRS^{S345}. Sample #1-#5 show tumors from mice treated with vehicle control, and #6-#10 show those treated with BVD. The experiments were repeated three times independently with similar results obtained (**b**).

Supplementary Fig.2

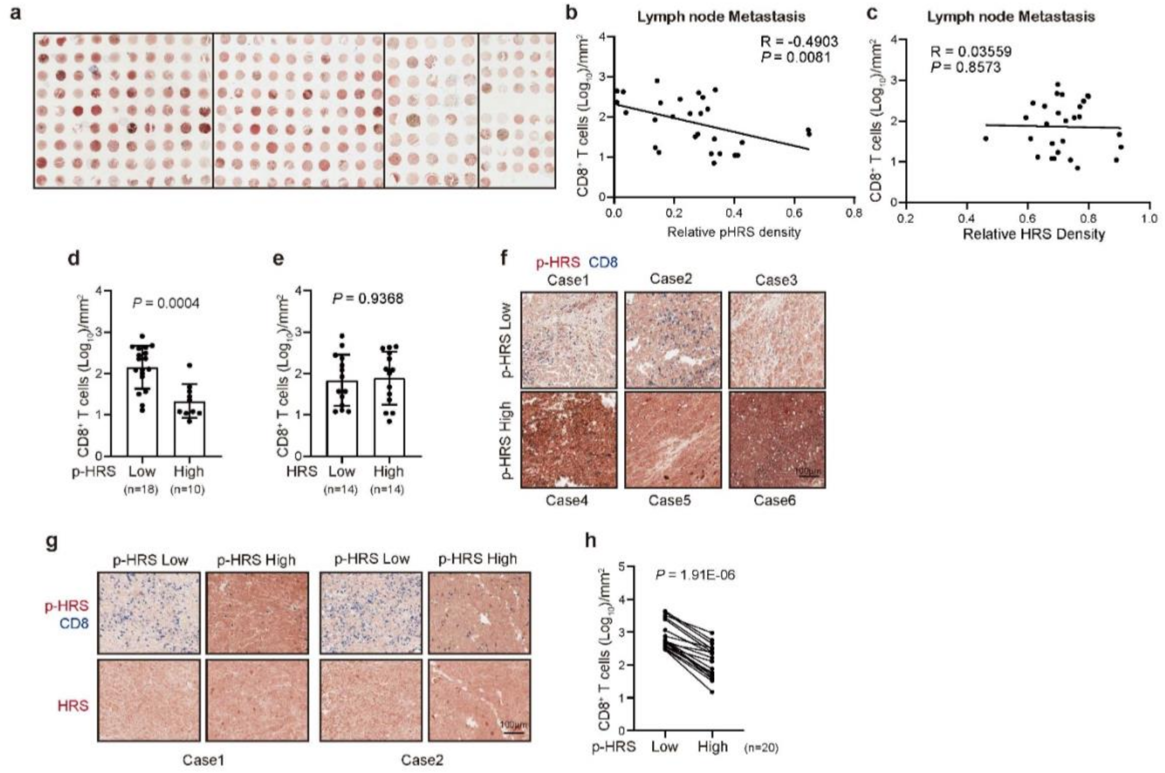

Supplementary Fig. 2. The level of HRS^{S345} phosphorylation is inversely correlated with CD8^{+} cell infiltration in melanoma.

a, p-HRS^{S345} staining in a tissue microarray with 217 primary malignant melanomas and 28 lymph node metastatic tumors. **b** and **c**, Correlation of p-HRS^{S345} (**b**) or total HRS (**c**) with CD8^{+} TILs in lymph node metastatic tumors in tissue microarray (n=28). **d**, The numbers of CD8^{+} TILs in lymph node metastatic tumor with low or high p-HRS^{S345} levels (p-HRS-low group, n=18; p-HRS-high group, n=10). **e**, The numbers of CD8^{+} TILs in lymph node metastatic tumors with low or high total HRS expression levels (HRS-low group, n=14; HRS-high group, n=14). **f**, Representative images of IHC staining of p-HRS^{S345} and CD8^{+} TILs in lymph node metastatic tumors in **d**. **g**, Representative IHC images showing p-HRS^{S345} and total HRS levels and CD8^{+} T cells in different areas of the tumors from two patients (total 20 patients). The upper and lower panels show the same area of the tumor. **h,** The numbers of CD8^{+} TILs in areas with high or low p-HRS^{S345} expression levels within the human melanoma tissues (n=20). 2-3 fields per group were counted in same patient. Data represent mean ± s.d. Statistical analyses were performed using Spearman correlation (**b, c**), two-tailed Mann-Whitney *U*-test (**d, e**) and two-tailed Wilcoxon test (**h**).

Supplementary Fig. 3

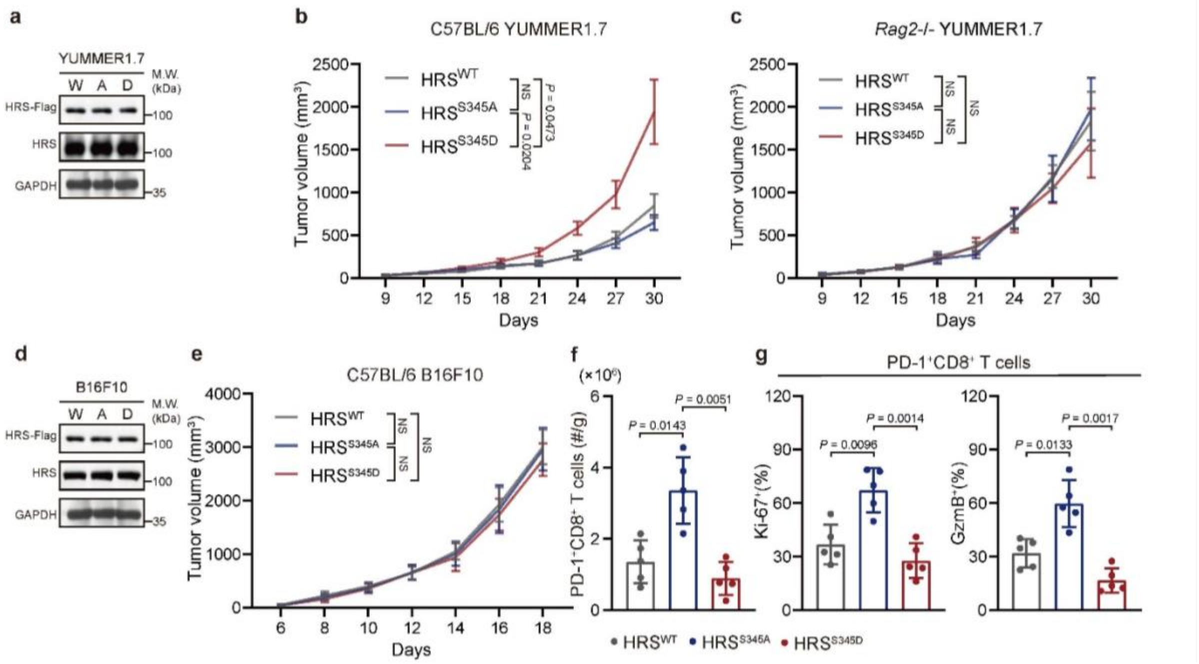

Supplementary Fig. 3. HRS phosphorylation suppresses CD8⁺ TILs *in vivo.*

a, Immunoblot analysis of Flag-tagged HRS variants and total HRS in YUMMER1.7 cell lines expressing Flag-tagged HRS^{WT} ("W"), HRS^{S345A} ("A") or HRS^{S345D} ("D"). GAPDH was used as a loading control. **b**, The growth of YUMMER 1.7 tumor expressing different HRS variants in C57BL/6 mice (n=5). **c**, The growth of YUMMER 1.7 tumor expressing different HRS variants in *Rag2*$^{-/-}$ mice (n=5). **d**, Immunoblot analysis of Flag-tagged HRS variants and total HRS in B16F10 cell lines. GAPDH was used as a loading control. **e**, The growth of B16F10 tumors expressing HRS variants in C57BL/6 mice (n=5). **f**, Bar graphs showing the numbers of PD-1^{+}CD8^{+} TILs (normalized to tumor weights) from the B16F10 cell tumors in **e**. **g**, Percentages of Ki-67^{+} (left) and Granzyme B^{+} (right) cells of PD-1^{+} CD8^{+} T cells in tumors in **e**. The experiments were repeated three times independently with similar results obtained (**a-g**). Data represent mean ± s.d. Statistical analyses were performed using two-way ANOVA (**b, c, e**) or one-way ANOVA (**f, g**). Tukey's test was used after ANOVA where multiple experimental groups were involved.

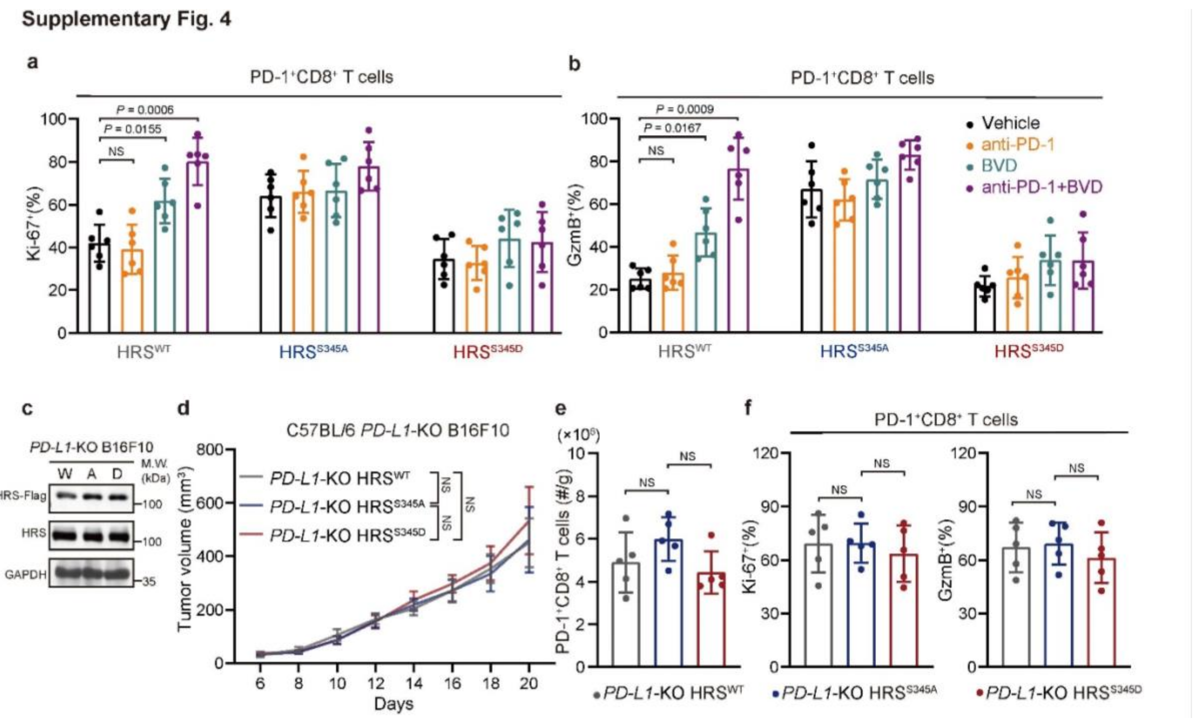

Supplementary Fig. 4. PD-L1 mediates the suppressive effect of p-HRS^{S345} on CD8^{+} cell infiltration.

a-b, Scatter-bar graphs depicting the percentages of Ki-67^{+} (**a**) and Granzyme B^{+} (**b**) in intratumor PD-1^{+}CD8^{+} T cells in tumors described in Fig. 2 **d**, **e** and **f** (n=6). **c**, The levels of HRS-Flag and total HRS in PD-L1 knockout (KO) B16F10 cell lines expressing Flag-tagged HRS wild type("W"), S345A("A") or S345D("D"). **d**, The growth of *PD-L1*-KO B16F10 tumor expressing Flag-tagged HRS^{WT} or mutants in C57BL/6 mice (n=5). **e**, Scatter-bar graphs showing the numbers of intratumor PD-1^{+}CD8^{+} T cells (normalized to tumor weights) in tumors in **d**. **f**, Percentages of Ki-67^{+} and Granzyme B^{+} cells in of PD-1^{+}CD8^{+} T cells in tumors in **d**. The experiments were repeated three times independently with similar results obtained (**a-f**). Data represent mean ± s.d. Statistical analyses were performed using one-way ANOVA (**a, b, e, f**) or two-way ANOVA (**d**). Tukey's test was used after ANOVA where multiple experimental groups were involved.

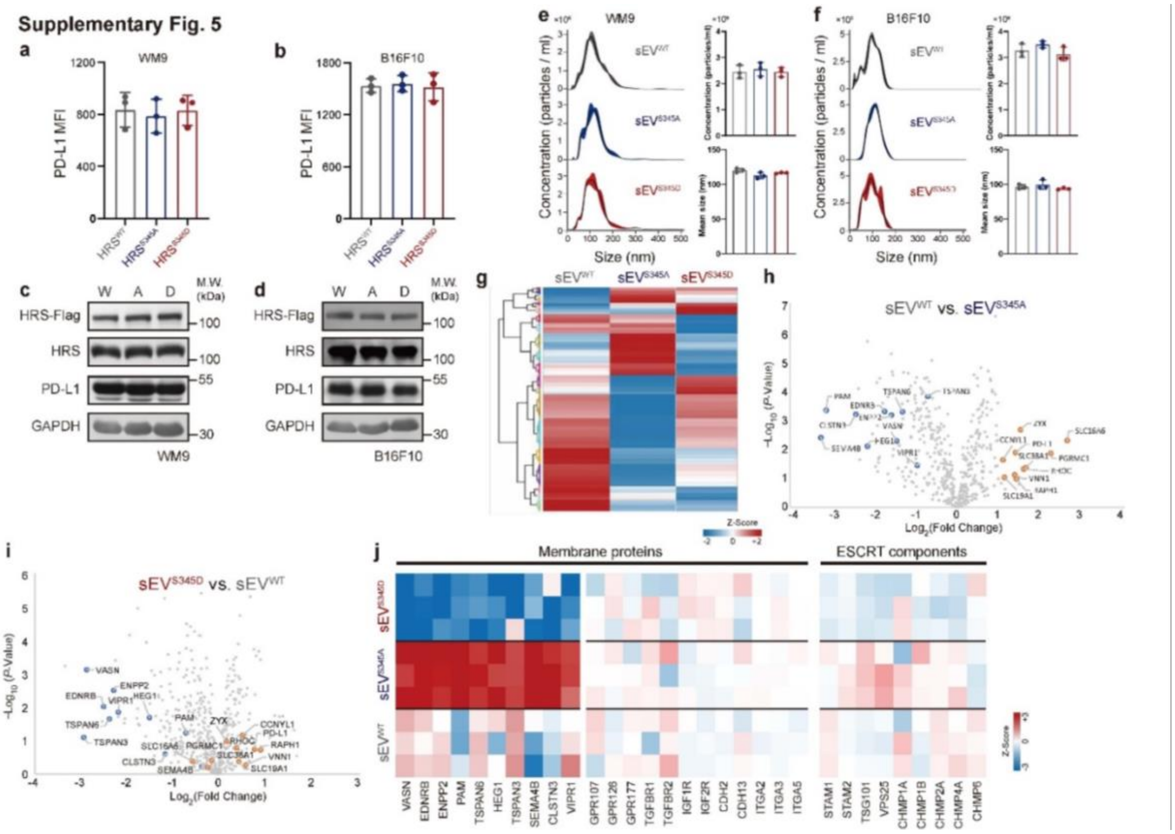

Supplementary Fig. 5. HRS phosphorylation selectively enriches a subgroup of proteins on sEV.

a-b, Mean fluorescence intensity (MFI) of PD-L1 on the surface of WM9 (**a**) and B16F10 (**b**) cells expressing different HRS variants. **c-d**, The levels of HRS-Flag, PD-L1 and total HRS in WM9 (**c**) and B16F10 (**d**) cells expressing Flag-tagged HRS variants. **e-f**, Single-particle tracking analysis of sEVs purified from the conditioned medium of WM9 (**e**) and B16F10 (**f**) cells expressing HRS variants. The X-axis indicates the diameters of the sEVs, and the Y-axis indicates the number of sEVs per ml. **g**, Hierarchical clustering showing differential sEV protein profiles among indicated groups. **h-i**, Volcano plots showing membrane proteins quantified by LC-MS/MS that are changed in sEVs derived from WM9 cells expressing HRS^{WT} and HRS^{S345A} (**h**) or sEVs derived from WM9 cells expressing HRS^{S345D} and HRS^{WT} (**i**) The 20 proteins having the largest fold-differences in sEVs derived from WM9 cells expressing HRS^{S345D} and HRS^{S345A} are labeled for comparison (See Fig. 3b). (**j**) Heatmap showing membrane proteins on sEV and the ESCRT components in indicated groups. The experiments were repeated three (**a-d**) and five (**e-f**) times independently with similar results obtained. Statistical analyses were performed using permutation-based FDR (**h, i**) or one-way ANOVA (**a, b, e, f**). Tukey's test was used after ANOVA where multiple experimental groups were involved.

Supplementary Fig. 6

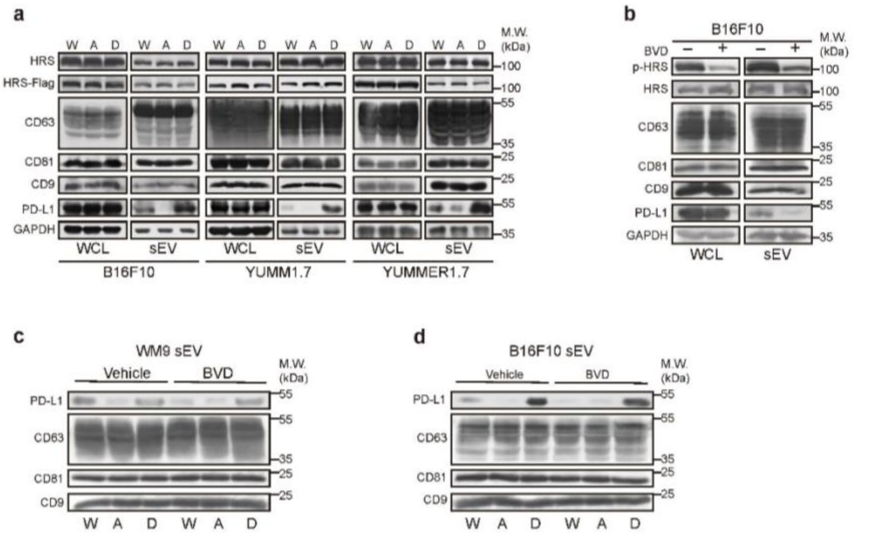

Supplementary Fig. 6. HRS phosphorylation enriches PD-L1 in sEVs.

a, Immunoblot analysis of HRS, HRS-Flag, PD-L1, and exosome marker proteins (CD63, CD81, and CD9) in whole cell lysate ("WCL") and purified sEVs from murine melanoma cell lines (B16F10, YUMM1.7, YUMMER1.7) expressing HRS^{WT} or mutants. The same amount of proteins were loaded. **b**, Immunoblot analysis of indicated proteins in whole cell lysate or purified sEVs from B16F10 cells with or without BVD-523 treatment. The same amount proteins were loaded. **c-d**, Immunoblot analysis of indicated protein in sEVs from WM9 (**c**) or B16F10 (**d**) cells expressing HRS variants with or without BVD-523 treatment. The same amount of proteins of were loaded. The experiments were repeated three times independently with similar results obtained (**a-d**).

Supplementary Fig.7

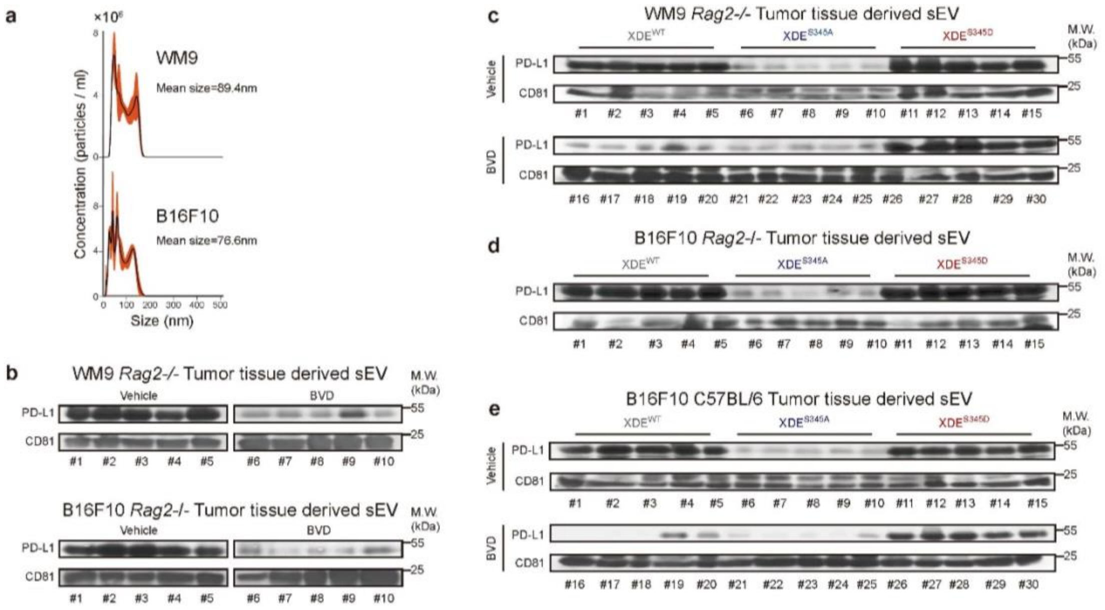

Supplementary Fig. 7. HRS phosphorylation enriches PD-L1 in sEVs *in vivo.*

a, Characterization of TTDEs from WM9 and B16F10 xenografts in *Rag2*$^{-/-}$ mice by nanoparticle tracking analysis. The X-axis indicates the diameters of the TTDEs, and the Y-axis indicates the numbers of TTDEs per ml. **b**, Western blot analysis of PD-L1 in TTDEs derived from WM9 (upper) and B16F10 (lower) tumors in *Rag2*$^{-/-}$ mice treated with vehicle or BVD-523 (n=5). The exosomes marker CD81 was used as a control. **c**, Western blot analysis of PD-L1 in TTDEs derived from WM9 tumors expressing HRS variants in *Rag2*$^{-/-}$ mice treated with vehicle or BVD-523 (n=5). **d**, Western blot analysis of PD-L1 in TTDEs derived from B16F10 tumors expressing HRS variants in *Rag2*$^{-/-}$ mice (n=5). **e**, Western blot analysis of PD-L1 in TTDEs from B16F10 tumors expressing wild type or mutant HRS in C57BL/6 mice treated with vehicle or BVD-523 (n=5). The same amounts of proteins were loaded in the western blot above. The experiments were repeated five (**a**) and three (**b-e**) times and independently with similar results obtained.

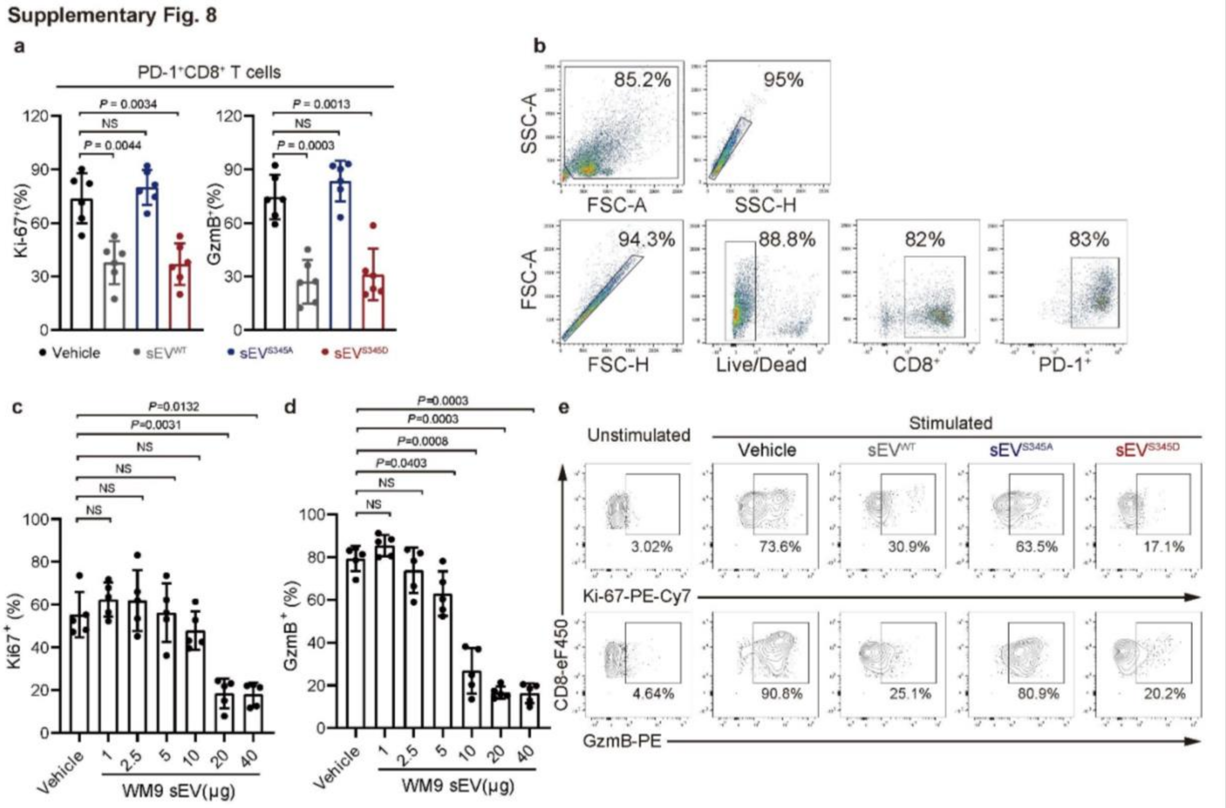

Supplementary Fig. 8. Suppression of human CD8$^+$ T cells by sEVs derived from melanoma cells.

a, Percentages of Ki-67$^+$ (left) and Granzyme B$^+$ (right) expression of PD-1$^+$ CD8$^+$ T cells in tumors described in Fig. 4a (n=6 for each group). **b**, Representative contour plots showing the general gating strategy used to sort PD-1$^+$ CD8$^+$ T cells from humans and mice. **c, d**, Percentages of Ki-67$^+$ and Granzyme B$^+$ cells of PD-1$^+$ CD8$^+$ T cells treated with vehicle or different concentrations of sEVs derived from WM9 cells (n=5). **e,** Representative contour plots of human peripheral CD8 T cells (stimulated with anti-CD3/CD28 antibodies) examined for the expression of Ki-67 (top) and Granzyme B (GzmB) (bottom) after indicated treatments. Data represent mean ± s.d. Statistical analyses were performed using one-way ANOVA (**a, c, d**). Tukey's test was used after ANOVA where multiple experimental groups were involved.

Supplementary Fig. 9

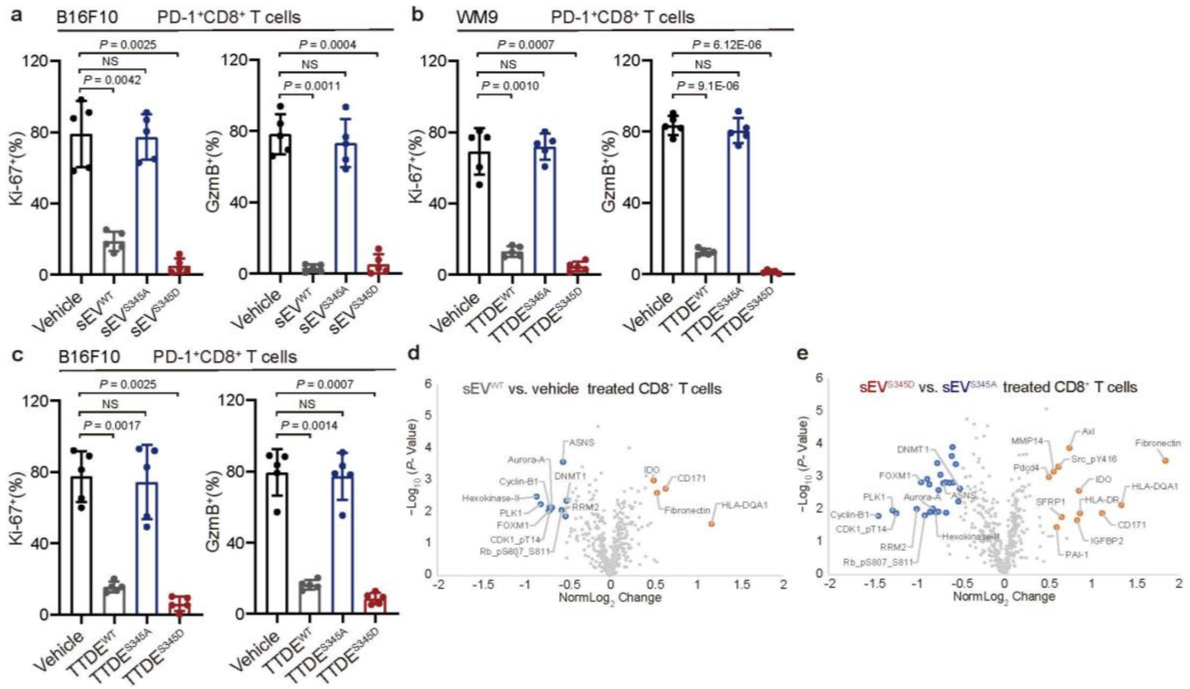

Supplementary Fig. 9. Suppression of human CD8$^+$ T cells by sEVs derived from cells with HRS phosphorylation.

a, Percentages of Ki-67$^+$ and Granzyme B$^+$ cells of PD-1$^+$CD8$^+$ T cells treated with vehicle or sEVs derived from B16F10 cells expressing different HRS variants (n=5). **b**, Percentages of Ki-67$^+$ and Granzyme B$^+$ cells of PD-1$^+$CD8$^+$ T cells treated with vehicle or TTDEs from WM9 tumors expressing different HRS variants (n=5). **c**, Percentages of Ki-67$^+$ and Granzyme B$^+$ cells of PD-1$^+$CD8$^+$ T cells treated with vehicle or TTDEs from B16F10 tumors expressing different HRS variants (n=5). **d**, Volcano plot showing the proteins that are changed in human CD8$^+$ T cells treated with sEV^{WT} from WM9 cells compared to vehicle control. **e**, Volcano plots showing the proteins that are changed in human CD8$^+$ T cells treated with sEV^{S345D} compared to sEV^{S345A} derived from WM9 cells. The most different protein is labeled blue (decreased) or orange (increased) in **d** and **e** based on Normlog$_2$ change>0.5 and P<0.05. Data represent mean ± s.d. Statistical analyses were performed using one-way ANOVA (**a-c**) or two-tailed Student's t test (**d, e**). The Tukey's test was used after ANOVA where multiple experimental groups were involved.

Supplementary Fig. 10

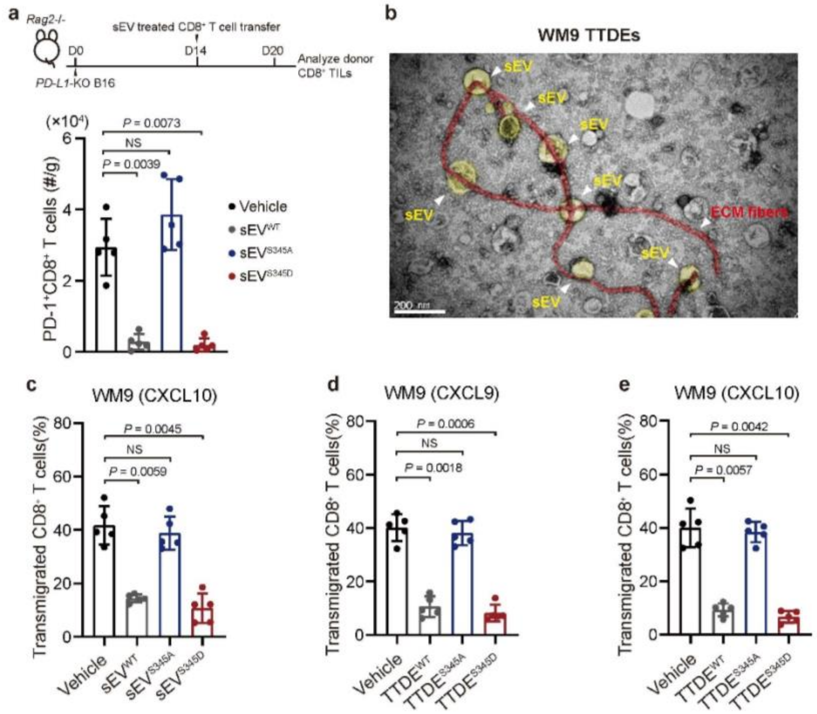

Supplementary Fig. 10. Inhibition of human CD8$^+$ T cell migration by sEVs and TTDEs derived from cells with HRS phosphorylation.

a, CD8$^+$ T cells were pretreated with vehicle or sEVs from B16F10 expressing HRS variants and transferred to *Rag*2$^{-/-}$ mice bearing *PD-L1*-KO B16F10 tumors at Day 14, shown as the experimental scheme for adoptive CD8$^+$ T cells co-transfer system (See detail in *METHODS*). The numbers of adopted PD-1$^+$ CD8$^+$ T cells in tumors were determined (normalized to tumor weight) at Day 20 (n=5). **b**, Transmission electron microscopy images of TTDEs isolated from WM9 xenografts established in *Rag2*$^{-/-}$ mice. The sEVs (highlighted yellow) associated with ECM fiber (red) were indicated by arrowhead. **c,** Percentages of human CD8$^+$ T cells migrated through the transwells induced by CXCL10 (n=5). The transwells were pretreated with or without indicated sEVs derived from WM9 cells. **d-e,** Percentages of CD8$^+$ T cells migrated through the transwells induced by CXCL9 or CXCL10 (n=5). The transwells were pretreated with or without indicated TTDEs derived from WM9 tumors. The experiments were repeated three times independently with similar results obtained (a-e). Data represent mean ± s.d. Statistical analyses were performed using one-way ANOVA (**a, c, d, e,**). Tukey's test was used after ANOVA where multiple experimental groups were involved.

Supplementary Fig. 11

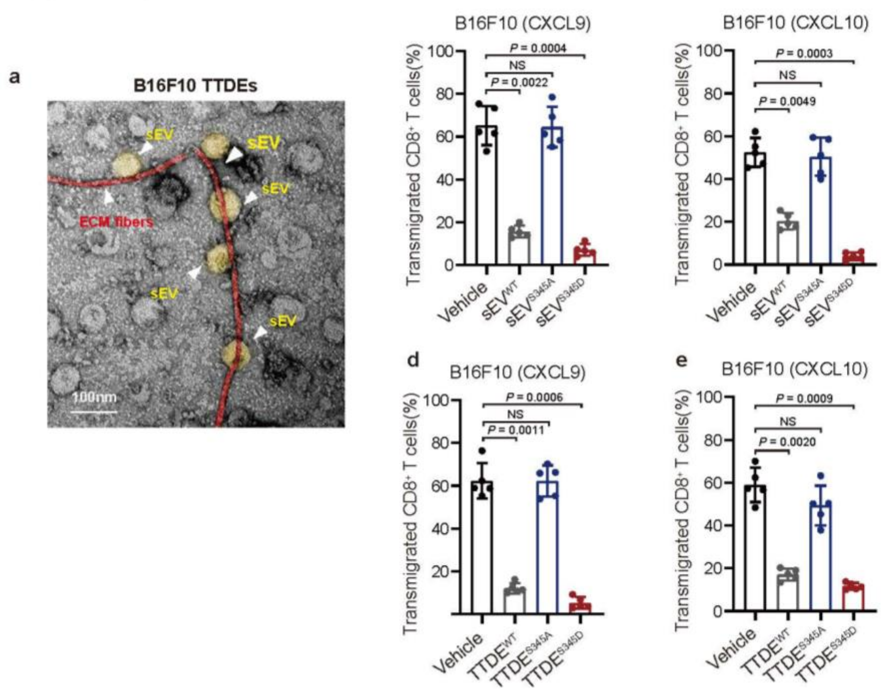

Supplementary Fig. 11. Inhibition of mouse CD8$^+$ T cell migration by sEVs derived from cells with HRS phosphorylation.

a, Transmission electron microscopy images of XDEs from B16F10 xenografts (*Rag*2$^{-/-}$ mice). The sEVs (highlighted yellow) associated with ECM fiber (red) were indicated by arrowhead. The experiments were repeated three times independently with similar results obtained. **b-e**, Transwells were pre-incubated with or without indicated sEVs from B16F10 cells (**b, c**) or TTDEs from B16F10 tumors (**d, e**). Percentages of transmigrated cells induced by CXCL9 (**b, d**) or CXCL10 (**c, e**) were determined. n=5. Data represent mean ± s.d. Statistical analyses were performed using one-way ANOVA (**b-e**). Tukey's test was used after ANOVA where multiple experimental groups were involved.

Supplementary Fig.12

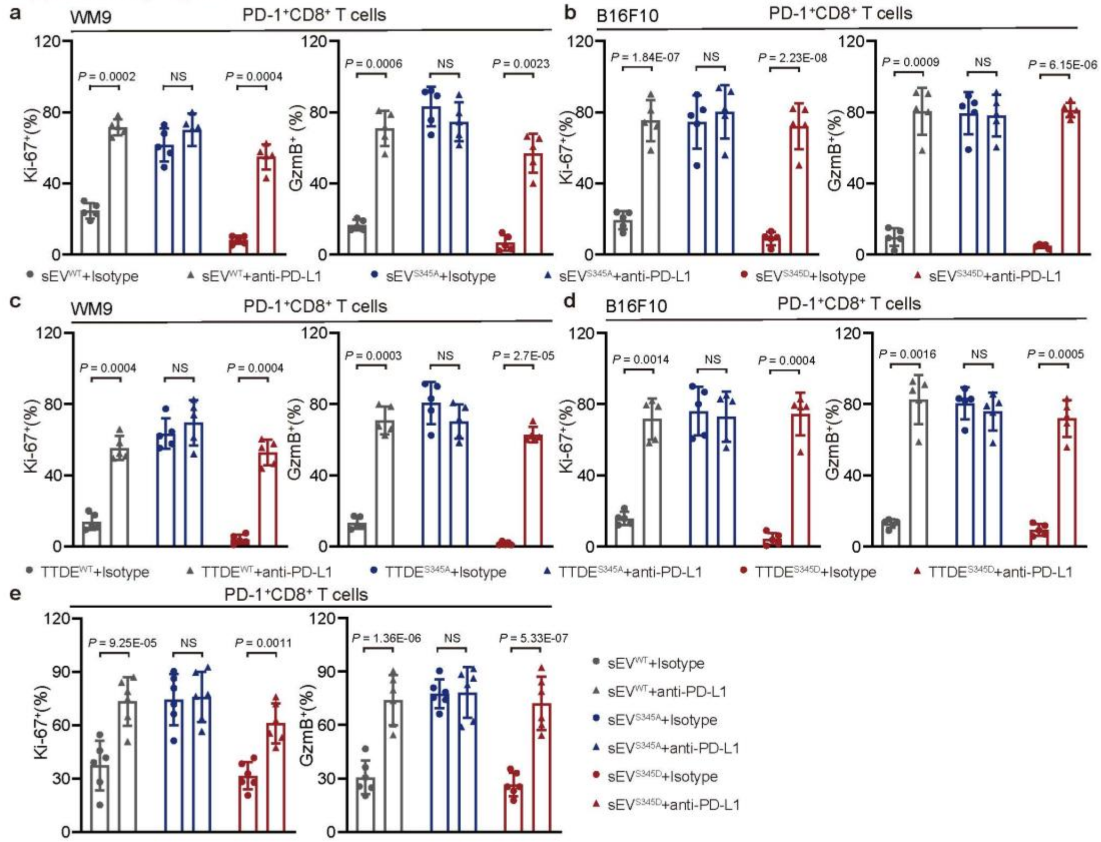

Supplementary Fig. 12. PD-L1 is required for CD8$^+$ T cell inhibition by sEVs derived from cells with HRS phosphorylation.

a-**b**, human (**a**) and mouse CD8$^+$ T cells (**b**) were treated with or without indicated sEVs from WM9 or B16F10 cells. The sEVs were preincubated with IgG control or anti-PD-L1 antibodies. The percentages of Ki-67$^+$ or Granzyme B$^+$ cells of PD-1$^+$CD8$^+$ T cells were accessed by flow cytometry (n=5). **c**-**d**, human (**c**) or mouse CD8$^+$ T cells (**d**) were treated with or without TTDEs from WM9 or B16F10 tumors. The TTDEs were preincubated with IgG control or anti-PD-L1 antibodies. The percentages of Ki-67$^+$ or Granzyme B$^+$ cells in PD-1$^+$CD8$^+$ T cells were accessed by flow cytometry (n=5). **e**, The percentages of Ki-67$^+$ (left) and Granzyme B$^+$ cells (right) of PD-1$^+$CD8$^+$ T cells in tumors described in Fig. 5**c** (n=6). The experiments were repeated three times independently with similar results obtained. Data represent mean ± s.d. Statistical analyses were performed using two-way ANOVA (**a**-**e**). Sidak's test was used after ANOVA where multiple experimental groups were involved.

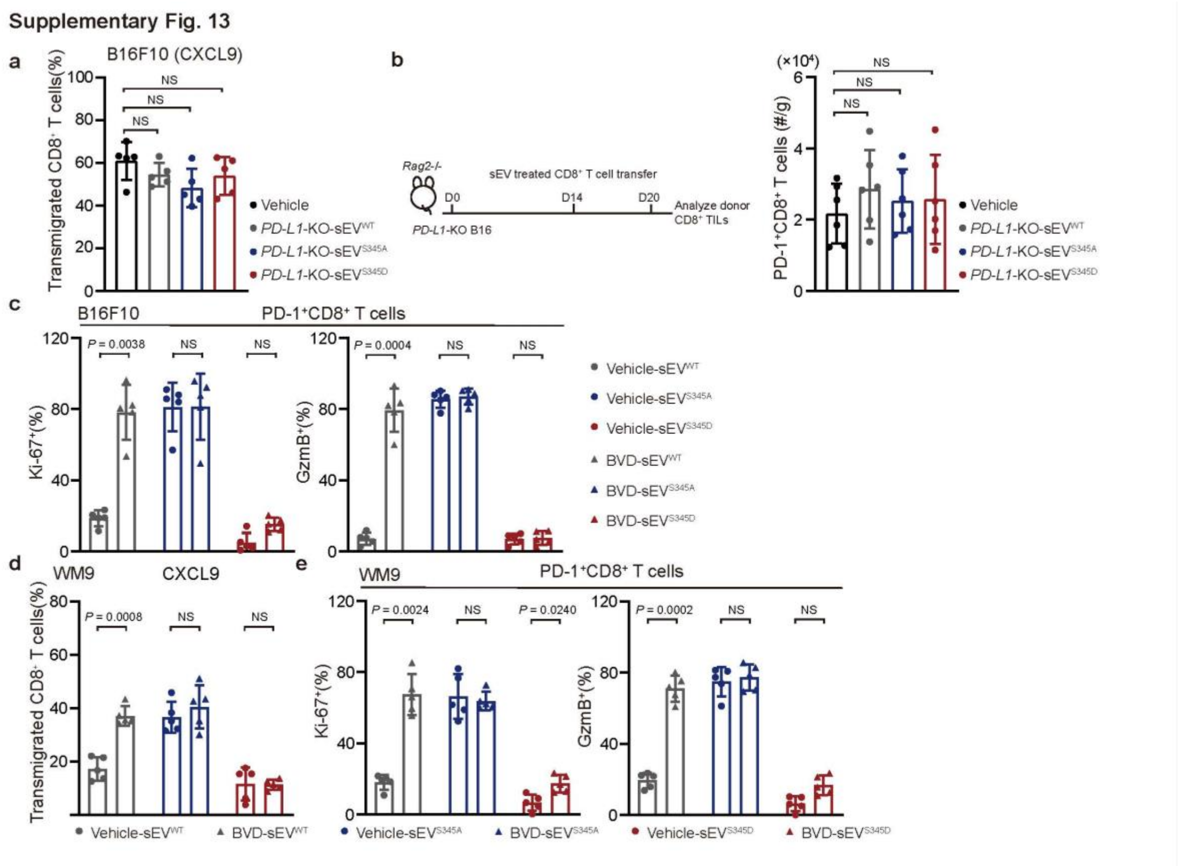

Supplementary Fig. 13. ERK inhibition attenuated the suppressive effect of sEVs.

a, Transwells were pretreated with sEVs from *PD-L1*-KO B1F10 cells expressing different HRS variants. Percentages of transmigrated mouse CD8$^+$ T cells were calculated (n=5). **b**, CD8$^+$ T cells were pretreated with vehicle or sEVs from *PD-L1*-KO B16F10 cells expressing HRS variants and transferred to *Rag*2$^{-/-}$ mice bearing *PD-L1*-KO B16F10 tumors at Day 14 (left), shown as the experimental scheme. The number of PD-1$^+$CD8$^+$ T cells in tumors were determined at Day 20 (n=6) (right). The experiments were repeated two times independently with similar results obtained. **c**, The percentages of Ki-67$^+$ (left) and Granzyme B$^+$ (right) cells of mouse PD-1$^+$CD8$^+$ T cells treated with sEVs from B16F10 cells with or without BVD-523 (n=5). **d**, Transwells were pretreated with sEVs from WM9 cells with or without BVD-523. Percentages of transmigrated human CD8$^+$ T cells were calculated (n=5). **e**, The percentages of Ki-67$^+$ (left) and Granzyme B$^+$ (right) cells of human PD-1$^+$CD8$^+$ T cells treated with sEVs from WM9 cells with or without BVD-523 (n=5). Data represent mean ± s.d. Statistical analyses were performed using one-way ANOVA (**a-b**) and two-way ANOVA (**c-e**). The Tukey's test (**a**, **b**) and Sidak's (**c**, **d**) were used after ANOVA where multiple experimental groups were involved.

Supplementary Fig. 14

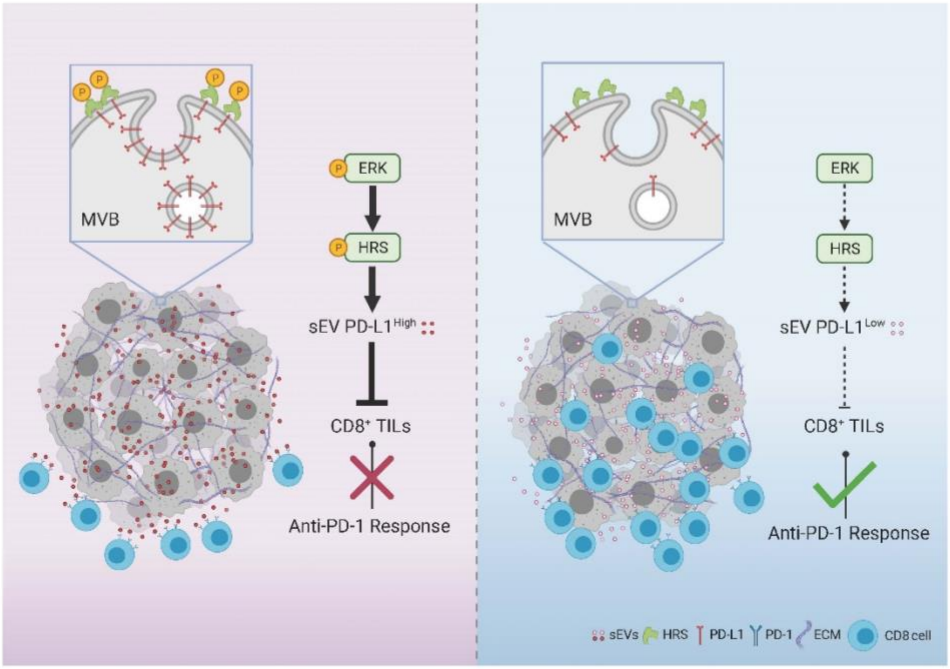

Supplementary Fig. 14. Proposed molecular mechanism for HRS phosphorylation by ERK and its inhibitory effect on CD8^{+} T cell infiltration into tumors. See *DISCUSSION* for details.

Supplementary Table 1. Information of antibodies

Antibody	Provider	Application (dilution)	Identifier
Anti-DYKDDDDK Tag	Cell Signaling Technology	WB(1:500), IF(1:500)	Cat#: 14793
Anti-Flag	Sigma	WB(1:1000), IF(1:500)	Cat#: F1804
Anti-HRS	Cell Signaling Technology	WB(1:400)	Cat#: 15087
Anti-HRS	Santa Cruz	IHC(1:200)	Cat#: 271925
Anti-Phospho-p44/42 MAPK(Erk1/2)	Cell Signaling Technology	WB(1:500)	Cat#: 4370
Anti-p44/42 MAPK(Erk1/2)	Cell Signaling Technology	WB(1:500)	Cat#: 4695
Anti-GAPDH	Cell Signaling Technology	WB(1:500)	Cat#: 5174
Anti-Phospho-MAPK Substrates	Cell Signaling Technology	WB(1:500)	Cat#: 2325
Anti-Phospho-MAPK Substrates	Cell Signaling Technology	WB(1:500)	Cat#: 14378
Anti-human CD8α	Cell Signaling Technology	IHC(1:200)	Cat#: 85336S
Anti-pHRS	Genscript	IHC(1:200), WB(1:500)	Customized
Anti-human PD-1	Cell Signaling Technology	IHC(1:500)	Cat#: 86163
Anti-human CD63	Abcam	WB(1:500)	Cat#: ab134045
Anti-mouse CD63	Abcam	WB(1:500)	Cat#: ab217345
Anti-human CD81	Cell Signaling Technology	WB(1:500)	Cat#: 10037
Anti-mouse CD81	Cell Signaling Technology	WB(1:500)	Cat#: 56039
Anti-human CD9	Cell Signaling Technology	WB(1:500)	Cat#: 13403
Anti-mouse CD9	Abcam	WB(1:500)	Cat#: ab92726
Anti-human PD-L1	Lab of Haidong Dong	Blocking(1:5), IF(1:500)	PMID: 21355078
Anti-human PD-L1	Cell Signaling Technology	IF(1:400), FCM(1:500)	Cat#: 86744
Anti-human PD-L1	Cell Signaling Technology	WB(1:500)	Cat#: 15165
Anti-mouse PD-L1	Abcam	WB(1:500)	Cat#: ab213480
Anti-E-Cadherin	Cell Signaling Technology	WB(1:500)	Cat#: 14472
Anti-STAM	Thermo Fisher Scientific	WB(1:300)	Cat#: 710512
Mouse IgG isotype control	BioLegend	Blocking(1:5)	Cat#: 401404
Anti-mouse PD-L1	Bio X Cell	Blocking(1:5)	Cat#: BE0101
Rat IgG2b isotype control	Bio X Cell	Blocking(1:10)	Cat#: BE0090
Anti-mouse PD-1	Bio X Cell	Blocking(1:10)	Cat#: BE0146
Anti-human CD3	Biolegend	FCM(1:500)	Cat#: 317340
Anti-human PD-L1	BioLegend	FCM(1:500)	Cat#: 329706
Anti-human PD-L1	BD Biosciences	FCM(1:500)	Cat#: 558065
Anti-human CD8	Invitrogen eBioscience	FCM(1:500)	Cat#: 48-0088-42
Anti-human PD-1	BioLegend	FCM(1:500)	Cat#: 329904
Anti-human Ki-67	BD Biosciences	FCM(1:300)	Cat#: 561283
Anti-mouse PD-1	BioLegend	FCM(1:500)	Cat#: 109110
Anti-human Granzyme B	Life Technologies	FCM(1:300)	Cat#: GRB04

Antibody	Provider	Application (dilution)	Identifier
Anti-mouse Ki-67	BioLegend	FCM(1:300)	Cat#: 652420
Anti-mouse Ki-67	BioLegend	FCM(1:300)	Cat#: 652410
Anti-mouse Ki-67	BioLegend	FCM(1:300)	Cat#: 652426
Anti-mouse Granzyme B	BioLegend	FCM(1:300)	Cat#: 12-8898-82
Anti-mouse Granzyme B	BioLegend	FCM(1:300)	Cat#: 515406
Anti-mouse CD3	BioLegend	FCM(1:500)	Cat#: 100204
Anti-mouse CD8a	eBioscience	FCM(1:500)	Cat#: 48-0081-82
Anti-mouse CD8a	BioLegend	FCM(1:500)	Cat#: 100714
Anti-Ghost Dye	Cell Signaling Technology	FCM(1:500)	Cat#: 49826
Anti-Ghost Dye	Cell Signaling Technology	FCM(1:500)	Cat#: 59863
Anti-Ghost Dye	Cell Signaling Technology	FCM(1:500)	Cat#: 18452
Anti-mouse CD45.1	Biolegend	FCM(1:500)	Cat#: 110707
Anti-mouse CD45.2	Biolegend	FCM(1:500)	Cat#: 109805
Anti-CD3	Biolegend	Activ(1:200)	Cat#: 300402
Anti-CD28	Biolegend	Co-stim(1:250)	Cat#: 302934
Anti-CD3	Biolegend	Activ(1:200)	Cat#: 100340
Anti-CD28	Biolegend	Co-stim(1:250)	Cat#: 102116
Anti-rabbit IgG Alexa Fluor 568	Invitrogen	IF(1:500)	Cat#: A-11011
Anti-mouse IgG Alexa Fluor 488	Invitrogen	IF(1:500)	Cat#: A-21202
Anti-mouse IgG, HRP	Cell Signaling Technolog	WB(1:3000)	Cat#: 7076
Anti-rabbit IgG, HRP	Cell Signaling Technolog	WB(1:3000)	Cat#: 7074

WB: Western Blotting; IF: Immunofluorescence; FCM: Flow cytometry; IP: Immunoprecipitation; Activ: Activation; Co-stim: Costimulation.
