# Supplementary material for: HRS phosphorylation drives immunosuppressive exosome secretion and restricts CD8+ T-cell infiltration into tumors
Source: Nat Commun. 2022 Jul 14;13:4078. doi: 10.1038/s41467-022-31713-6 (PMC9283393; doi:10.1038/s41467-022-31713-6)
Supplement: Supplementary file 3 — Reporting Summary [file 41467_2022_31713_MOESM3_ESM.pdf]

nature portfolio

Corresponding author(s): Wei Guo

Last updated by author(s): 06/10/2022

Reporting Summary

Nature Portfolio wishes to improve the reproducibility of the work that we publish. This form provides structure for consistency and transparency in reporting. For further information on Nature Portfolio policies, see our Editorial Policies and the Editorial Policy Checklist.

Statistics

For all statistical analyses, confirm that the following items are present in the figure legend, table legend, main text, or Methods section.

n/a	Confirmed	
☐	☒	The exact sample size (*n*) for each experimental group/condition, given as a discrete number and unit of measurement
☐	☒	A statement on whether measurements were taken from distinct samples or whether the same sample was measured repeatedly
☐	☒	The statistical test(s) used AND whether they are one- or two-sided<br>*Only common tests should be described solely by name; describe more complex techniques in the Methods section.*
☐	☒	A description of all covariates tested
☐	☒	A description of any assumptions or corrections, such as tests of normality and adjustment for multiple comparisons
☐	☒	A full description of the statistical parameters including central tendency (e.g. means) or other basic estimates (e.g. regression coefficient) AND variation (e.g. standard deviation) or associated estimates of uncertainty (e.g. confidence intervals)
☐	☒	For null hypothesis testing, the test statistic (e.g. *F*, *t*, *r*) with confidence intervals, effect sizes, degrees of freedom and *P* value noted<br>*Give P values as exact values whenever suitable.*
☐	☒	For Bayesian analysis, information on the choice of priors and Markov chain Monte Carlo settings
☐	☒	For hierarchical and complex designs, identification of the appropriate level for tests and full reporting of outcomes
☐	☒	Estimates of effect sizes (e.g. Cohen's *d*, Pearson's *r*), indicating how they were calculated

Our web collection on statistics for biologists contains articles on many of the points above.

Software and code

Policy information about availability of computer code

Data collection	pFind3 3.1.6, pLabel 2.4.1, NIS-Elements-AR 5.21, NanoSight NTA 3.2, Perseus 1.6.2.3,FluorEssence™2.0, GraphPad Prism 8.0, Q-path0.2.3, FlowJo10.6.2, MaxQuant 1.6.7.0
Data analysis	Statistical analyses were performed with GraphPad Prism 8.0, LC-MS/MS were analyzed by Ingenuity Pathway Analysis and Principal Component Analysis by Perseus 1.6.2.3

For manuscripts utilizing custom algorithms or software that are central to the research but not yet described in published literature, software must be made available to editors and reviewers. We strongly encourage code deposition in a community repository (e.g. GitHub). See the Nature Portfolio guidelines for submitting code & software for further information.

Data

Policy information about availability of data

All manuscripts must include a data availability statement. This statement should provide the following information, where applicable:

- Accession codes, unique identifiers, or web links for publicly available datasets
- A description of any restrictions on data availability
- For clinical datasets or third party data, please ensure that the statement adheres to our policy

RPPA data are available from the NCBI Gene Expression Omnibus (GEO) under accession numbers GSE174270 [https://www.ncbi.nlm.nih.gov/geo/query/acc.cgi?acc=GSE174270]. The mass spectrometry proteomics data have been deposited to the MassIVE data repository with the accession number MSV000088846 [ftp://massive.ucsd.edu/MSV000088846/] and to the Proteome Xchange Consortium with the accession number PXD031715 [http://proteomecentral.proteomexchange.org/cgi/GetDataset?ID=PXD031715]. All data are included in the Supplemental Information or available from the authors upon reasonable requests, as are unique reagents used in this article. The raw numbers for charts and graphs are available in the Source Data file whenever possible.

March 2021

Field-specific reporting

Please select the one below that is the best fit for your research. If you are not sure, read the appropriate sections before making your selection.

- [x] Life sciences
- [ ] Behavioural & social sciences
- [ ] Ecological, evolutionary & environmental sciences

For a reference copy of the document with all sections, see nature.com/documents/nr-reporting-summary-flat.pdf

Life sciences study design

All studies must disclose on these points even when the disclosure is negative.

Sample size	For mouse studies, the sample size (n≥5/group) was determined based on our previous experience with the models to provide sufficient statistical power (Chen et al., Nature 2018).
Data exclusions	No data were excluded from analysis.
Replication	Each figure describes how many times each experiment had been repeated. All attempts to replicate the experiments were successful.
Randomization	Mice and other samples in experiments were allocated randomly to each treatment group.
Blinding	The assessment for patients was performed independently in a double-blinded fashion. For all mouse studies, the experiments were performed in a blinded fashion. Downstream analyses of mouse samples (immunofluorescence staining and flow cytometry) were performed in a blinded fashion, which means that the individuals performing the assays were not aware of the treatment groups until data analyses were completed. For experiments other than those involving patients and animals, the investigators were blinded to group allocation during data collection and/or analysis.

Reporting for specific materials, systems and methods

We require information from authors about some types of materials, experimental systems and methods used in many studies. Here, indicate whether each material, system or method listed is relevant to your study. If you are not sure if a list item applies to your research, read the appropriate section before selecting a response.

Materials & experimental systems

n/a	Involved in the study	
☐	☒	Antibodies
☐	☒	Eukaryotic cell lines
☒	☐	Palaeontology and archaeology
☐	☒	Animals and other organisms
☐	☒	Human research participants
☒	☐	Clinical data
☒	☐	Dual use research of concern

Methods

n/a	Involved in the study	
☒	☐	ChIP-seq
☐	☒	Flow cytometry
☒	☐	MRI-based neuroimaging

Antibodies

Antibodies used	Anti-DYKDDDDK Tag Cell Signaling Technology Cat#: 14793; Anti-Flag Sigma Cat#: F1804; Anti-HRS Cell Signaling Technology Cat#: 15087; Anti-HRS Santa Cruz Cat#: 271925; Anti-Phospho-p44/42 MAPK(Erk1/2) Cell Signaling Technology Cat#: 4370; Anti-p44/42 MAPK(Erk1/2) Cell Signaling Technology Cat#: 4695; Anti-GAPDH Cell Signaling Technology Cat#: 5174; Anti-Phospho-MAPK Substrates Cell Signaling Technology Cat#: 2325; Anti-Phospho-MAPK Substrates Cell Signaling Technology Cat#: 14378Anti-human CD8α Cell Signaling Technology Cat#: 85336S; Anti-pHRS Genscript Customized; Anti-human PD-1 Cell Signaling Technology Cat#: 86163; Anti-human CD63 Abcam Cat#: ab134045; Anti-mouse CD63 Abcam Cat#: ab217345; Anti-human CD81 Cell Signaling Technology Cat#: 10037; Anti-mouse CD81 Cell Signaling Technology Cat#: 56039; Anti-human CD9 Cell Signaling Technology Cat#: 13403; Anti-mouse CD9 Abcam Cat#: ab92726; Anti-human PD-L1 Lab of Haidong Dong PMID: 21355078; Anti-human PD-L1 Cell Signaling Technology Cat#: 86744; Anti-human PD-L1 Cell Signaling Technology Cat#: 15165; Anti-mouse PD-L1 Abcam Cat#: ab213480; Anti-E-Cadherin Cell Signaling Technology Cat#: 14472; Anti-STAM Thermo Fisher Scientific Cat#: 710512; Mouse IgG isotype control BioLegend Cat#: 401404; Anti-mouse PD-L1 Bio X Cell Cat#: BE0101; Rat IgG2b isotype control Bio X Cell Cat#: BE0090; Anti-mouse PD-1 Bio X Cell Cat#: BE0146; Anti-human CD3 Biolegend Cat#: 317340; Anti-human PD-L1 BioLegend Cat#: 329706; Anti-human PD-L1 BD Biosciences Cat#: 558065; Anti-human CD8 Invitrogen eBioscience Cat#: 48-0088-42; Anti-human PD-1 BioLegend Cat#: 329904; Anti-human Ki-67 BD Biosciences Cat#: 561283; Anti-human Granzyme B Life Technologies Cat#: GRB04; Anti-mouse PD-1 BioLegend Cat#: 109110; Anti-mouse Ki-67 BioLegend Cat#: 652420; Anti-mouse Ki-67 BioLegend Cat#: 652410; Anti-mouse Ki-67 BioLegend Cat#: 652426; Anti-mouse Granzyme B BioLegend Cat#: 12-8898-82; Anti-mouse Granzyme B BioLegend Cat#: 515406; Anti-mouse CD3 BioLegend Cat#: 100204; Anti-mouse CD8a eBioscience Cat#: 48-0081-82; Anti-mouse CD8a BioLegend Cat#: 100714; Anti-Ghost Dye Cell Signaling Technology Cat#: 49826; Anti-Ghost Dye Cell Signaling Technology Cat#: 59863; Anti-Ghost Dye Cell Signaling Technology Cat#: 18452 ; Anti-mouse CD45.1 Biolegend Cat#: 110707; Anti-mouse CD45.2 Biolegend Cat#: 109805; Anti-CD3 Biolegend Cat#: 300402; Anti-CD28 Biolegend Cat#: 302934; Anti-CD3 Biolegend Cat#: 100340;

	Anti-CD28 Biolegend Cat#: 102116; Anti-CD28 Biolegend Cat#: 102116; Anti-rabbit IgG Alexa Fluor 568 Invitrogen Cat#: A-11011; Anti-mouse IgG Alexa Fluor 488 Invitrogen Cat#: A-21202; Anti-mouse IgG, HRP-linked Antibody Cell Signaling Technology Cat#:7076; Anti-rabbit IgG, HRP-linked Antibody Cell Signaling Technology Cat#:7074. Dilutions for the antibodies are shown in Supplementary Table 1.
Validation	Validation statements for all primary antibody are provided on the manufacture's website. Antibodies were further validated by using positive and negative controls in our studies, or based on correct molecular masses.

Eukaryotic cell lines

Policy information about cell lines

Cell line source(s)	The A375 human melanoma and B16-F10 mouse melanoma cells were purchased from ATCC. The PD-L1 knock out B16F10 cell line was provided by Dr. Haidong Dong (Mayo Clinic). YUMM 1.7 and YUMMER 1.7 mouse melanoma cell lines were provided by Dr. Marcus Bosenberg (Yale). WM164 and WM9 were established in Dr. Meenhard Herlyn's lab (Wistar Institute).
Authentication	All of the cell lines used were authenticated by STR profiling.
Mycoplasma contamination	Mycoplasma testing was regularly conducted to assure that all cells used were mycoplasma free.
Commonly misidentified lines (See ICLAC register)	No commonly misidentified cell lines were used for this study.

Animals and other organisms

Policy information about studies involving animals; ARRIVE guidelines recommended for reporting animal research

Laboratory animals	6-8-week-old male C57BL/6 mice were housed under standard specific-pathogen-free (SPF) conditions. Mice were housed at 23± 2°C ambient temperature with 40% humidity and a 12 hr light/dark cycle (7 am on and 7pm off).
Wild animals	Study did not involve wild animals.
Field-collected samples	No samples were collected in Field.
Ethics oversight	All animal experiments were performed in accordance with protocols approved by the Institutional Animal Care and Use Committee of University of Pennsylvania.

Note that full information on the approval of the study protocol must also be provided in the manuscript.

Human research participants

Policy information about studies involving human research participants

Population characteristics	Blood samples from healthy male and female individuals were used in T cell experiments. There was no specific consideration/preference of covariate-relevant population characteristics for these individuals.
Recruitment	Healthy individuals were recruited by by the Human Immunology Core at the University of Pennsylvania, with no specific selection basis for blood donation used for T cell isolation.
Ethics oversight	Blood samples from human healthy donors were collected by the Human Immunology Core at the University of Pennsylvania with the approval from the ethics committee and institutional review board. Written consent was obtained from each healthy donor before blood collection. All experiments involving blood samples from healthy donors were performed in accordance with relevant ethical regulations.

Note that full information on the approval of the study protocol must also be provided in the manuscript.

Flow Cytometry

Plots

Confirm that:

- [x] The axis labels state the marker and fluorochrome used (e.g. CD4-FITC).
- [x] The axis scales are clearly visible. Include numbers along axes only for bottom left plot of group (a 'group' is an analysis of identical markers).
- [x] All plots are contour plots with outliers or pseudocolor plots.
- [x] A numerical value for number of cells or percentage (with statistics) is provided.

Methodology

Sample preparation	Single cell suspension of spleen was obtained by mechanical desegregation in FACS buffer (PBS + 2% FCS + 1mM EDTA). To obtain tumor-infiltrating cells, tumors were digested in RPMI (Gibco) supplemented with 0.5 mg/ml collagenase type IV (Gibco) and 0.1 mg/ml DNase I (Sigma) for 20 min at 37 °C.
Instrument	LSRII (BD Biosciences, San Jose, CA)
Software	FlowJo v10.0.7 (BD)
Cell population abundance	Sorting was not used in this study.
Gating strategy	Doublets were removed from total population using FSC-A and FSC-H. Total singlets were gated using FSC-A and SSC-A. Single staining were used for compensation caculation. Positive population were gated based on comparing unstained sample and single stained sample.

☒ Tick this box to confirm that a figure exemplifying the gating strategy is provided in the Supplementary Information.
